# Calibration Markers for Digital Templating in Total Hip Arthroplasty

**DOI:** 10.1371/journal.pone.0128529

**Published:** 2015-07-13

**Authors:** Christoph Kolja Boese, Philipp Lechler, Leonard Rose, Jens Dargel, Johannes Oppermann, Peer Eysel, Hansjörg Geiges, Jan Bredow

**Affiliations:** 1 Department of Orthopaedic and Trauma Surgery, University Hospital of Cologne, Cologne, Germany; 2 Department of Trauma, Hand and Reconstructive Surgery, University of Giessen and Marburg, Marburg, Germany; 3 Mathematical Institute, University of Cologne, Cologne, Germany; University of Sheffield, UNITED KINGDOM

## Abstract

Digital templating with external calibration markers is the standard method for planning total hip arthroplasty. We determined the geometrical basis of the magnification effect, compared magnification with external and internal calibration markers, and examined the influence on magnification of the position of the calibration markers, patient weight, and body mass index (BMI). A formula was derived to calculate magnification with internal and external calibration markers, informed by 100 digital radiographs of the pelvis. Intraclass correlations between the measured and calculated values and the strength of relationships between magnification, position and distance of calibration markers and height, weight, and BMI were sought. There was a weak correlation between magnification of internal and external calibration markers (r = 0.297–0.361; p < 0.01). Intraclass correlations were 0.882–1.000 (p = 0.000) for all parameters. There were also weak correlations between magnification of internal and external calibration markers and weight and BMI (r = 0.420, p = 0.000; r = 0.428, p = 0.000, respectively). The correlation between external and internal calibration markers was poor, indicating the need for more accurate calibration methods. While weight and BMI weakly correlated with the magnification of markers, future studies should examine this phenomenon in more detail.

## Introduction

Digital templating has become the standard method of preoperative planning for total hip arthroplasty, aiming to optimize component choice and positioning, and to minimize the risk of intra- and postoperative complications [1]. Calibration requires standardized radiographs of the pelvis to be compared with an object of known size, while conventional acetate templating relies on a fixed magnification [2]. The external calibration marker (ECM), usually a sphere, should be positioned at the height of the region of interest (ROI; i.e., the center of the hip joint) relative to the detector plate and central beam [1,2]. Positioning of the ECM is complicated by the difficulty of identifying the correct anatomic landmarks by palpation, and other patient-specific factors [3]. Patients may also find the requirement to position the ECM near the anus and genitals distressing or uncomfortable[4].

The geometrical principles of radiological magnification depend on the vertical and horizontal distance from the X-ray source and the form of the marker [5]. Previous studies investigating the precision of digital templating focused only on the effect of the vertical position of the ECM [2,4,6]. In practice, the ECM can be positioned laterally adjacent to the greater trochanter, or medially between the legs [2,3]. The magnifications of the ECM and the ROI are only identical when they are level in the horizontal plane and have the same distance from the central beam; thus the magnification factor can only be correct when the ECM is held between the legs.

The first objective of this study was to determine the geometrical basis of the magnification effect of objects in the X-ray beam. The second was to analyze the magnification of an ECM compared with an internal calibration marker (ICM, i.e., the head component of a hip arthroplasty), the latter being equivalent to the true magnification of the ROI. Third, the influences of the position of the ECM, patient weight, and body mass index (BMI) on the magnification factor were analyzed. Intra- and inter-observer reliabilities were assessed.

## Materials and Methods

### Geometric principles of radiographic magnification

**Fig 1:**
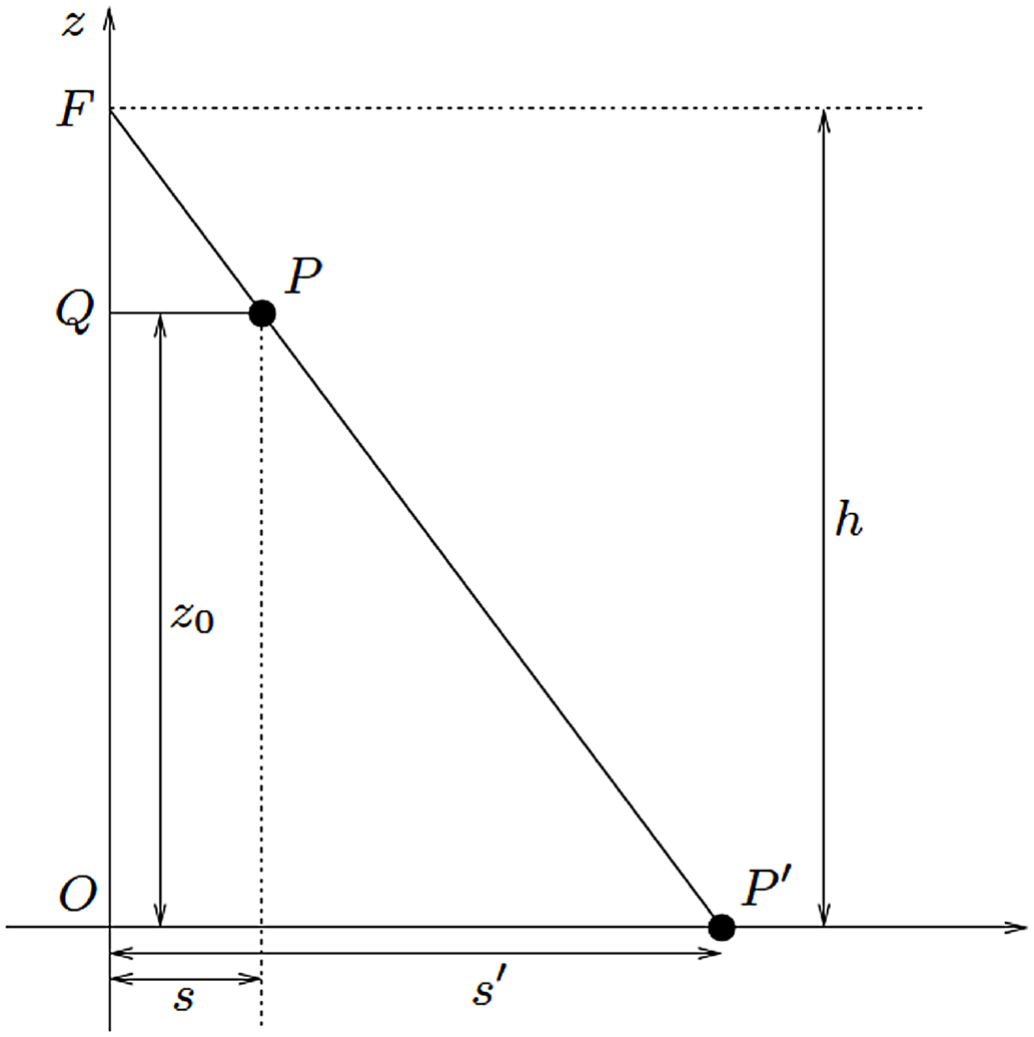
Projection of a point (or any flat object). The focus (F) of the X-ray beam is located at height (h) over the origin (O) of the xy-plane.

The projection of objects in the X-ray beam depends on the position of the object in the vertical and horizontal planes, while the plane of projection is the *xy*-plane (i.e., the detector) in a three-dimensional Cartesian coordinate system; the focus (*F*) of the X-ray beam is located at height (*h*) over the origin (*O*) of the *xy*-plane (Fig 1). Thus, the object is centered at a horizontal distance *x*_0_ (s) from the focus and at height *z*_0_ over the *xy*-plane.

### Projecting a disc

A point at height *z*_0_ and horizontal distance *s* from the focus is projected (*s’*) to the *xy*-plane according to the formula:

$$\;s^{\prime }=\;\frac{\left|FO\right|}{\left|FQ\right|}\;\cdot \text{s}=\;\frac{h}{h-z_{0}}\;\cdot \text{s}$$ (1)

Therefore, any flat object (i.e. a disc parallel to the *xy*-plane) is magnified by a factor (*m*):

$$m=\;\frac{h}{\left(h-\;z_{0}\right)}.$$ (2)

### Projecting a sphere

**Fig 2:**
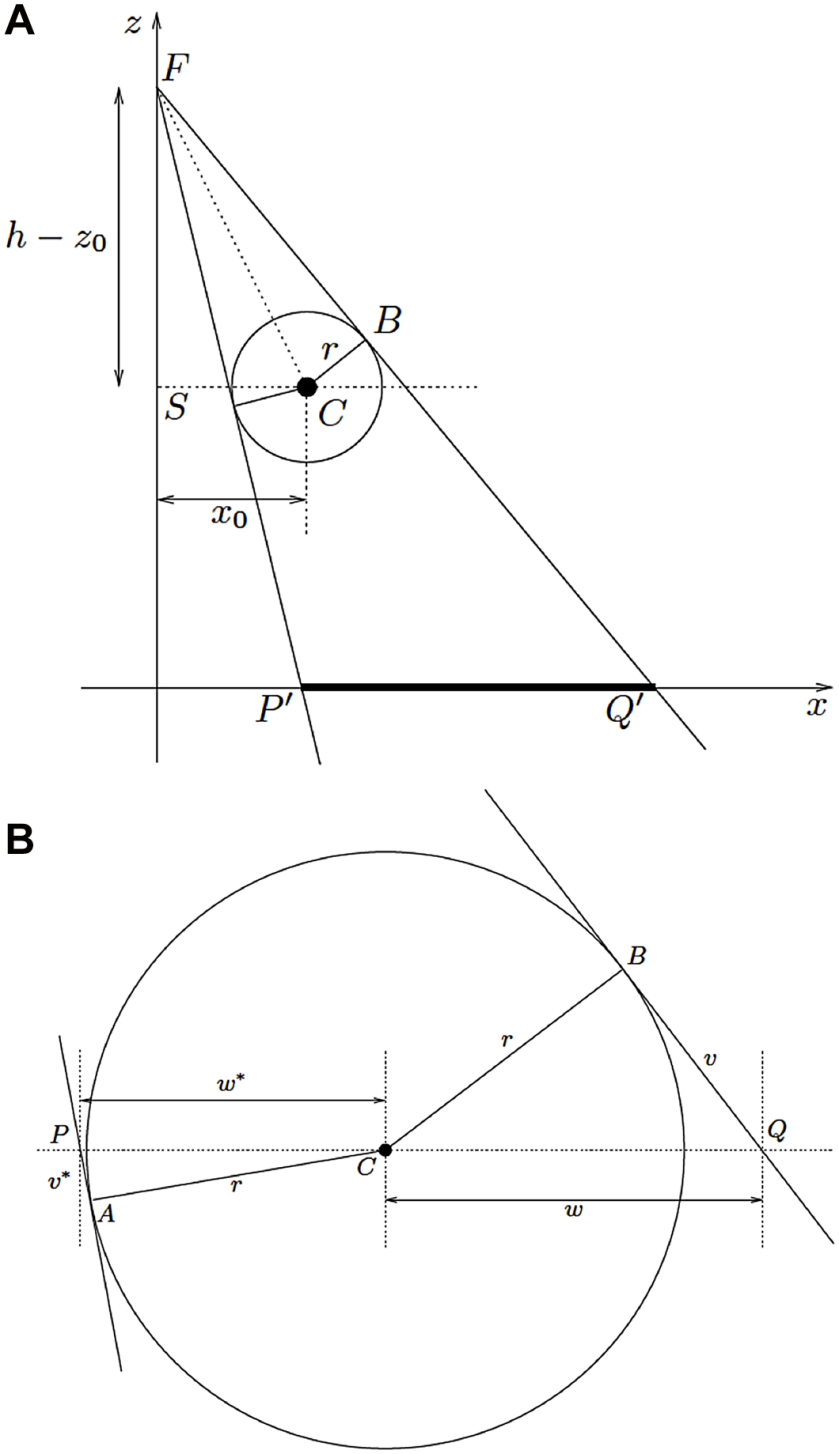
A. Projection of a sphere. The focus (F) of the X-ray beam is located at height (h) over the origin (O) of the xy-plane. A sphere with radius (r) and the center (C) shifted from the X-ray beam by *x*_*0*_ (S) is projected to the *xy*-plane (|P’Q’|). B. Detailed view of the projection of a sphere with radius (r). The center of the sphere (C) and the projected length |PC| (w*) and |PQ| (*w*) are shown. A and B are cutting points of the tangential X-ray beam and the sphere.

The projection of a spherical object of radius *r* in the X-ray beam results in an ellipse or circle (Fig 2A). Formulae for the minor and major axes of the ellipse were derived, but only the major axis is clinically relevant. To determine the projection of a sphere of radius *r* in the same way as a disc, it must first be transformed into a flat projection. Thus, the sum of the lengths w* and *w* requires definition (Fig 2B):

$$\left|PQ\right|=w^{*}+w=\;\frac{2r\left(h-\;z_{0}\right)\sqrt{x_{0}^{2}+{\left(h-\;z_{0}\right)}^{2}-\;r^{2}}}{{\left(h-z_{0}\right)}^{2}-\;r^{2}}.$$ (3)

Considered with argument 1, the major axis of a projected sphere is thus:

$$\left|P\prime Q\prime \right|=m\left|PQ\right|=\;\frac{2rh\sqrt{x_{0}^{2}+{\left(h-z_{0}\right)}^{2}-r^{2}}}{{\left(h-z_{0}\right)}^{2}-\;r^{2}}.$$ (4)

The detailed mathematical derivation can be found in the supplementary data (S1 Text).

The projected diameter of a sphere in the X-ray beam can be calculated for any position using formula 4. The theoretical magnification of a 28-mm-diameter sphere was calculated for various amounts of vertical (distance above the detector, *z*_0_) and horizontal displacement (distance from the central beam in the *xy*-plane, *x*_0_). The absolute size of the projection, the proportion of the overall magnification (as a percentage), and the magnitude of the horizontal shift alone are described.

### Analysis of radiographs

We identified 100 standing anteroposterior (AP) radiographs of the pelvis from the hospital picture archiving and communication system (PACS) in a retrospective search that spanned March 2012 to September 2014. Inclusion criteria were: (1) standing AP radiograph of the pelvis, (2) presence of a unilateral primary total hip arthroplasty to act as an ICM, (3) with complete documentation of subsequent implant type and size, and (4) identifiable complete depiction of the ECM.

All radiographs were acquired with a Philips DigitalDiagnost (Philips GmbH, Hamburg, Germany) with a tube-to-detector distance of 1100 mm. Patients stood with their feet internally rotated to neutralize the assumed anatomical anteversion of the hip and the beam was centered on the pubic symphysis. A spherical external calibration marker of 28 mm diameter was secured to the patient either medially between the legs or laterally on the thigh adjacent to the position at which the greater trochanter could be palpated. Images were stored digitally.

**Fig 3:**
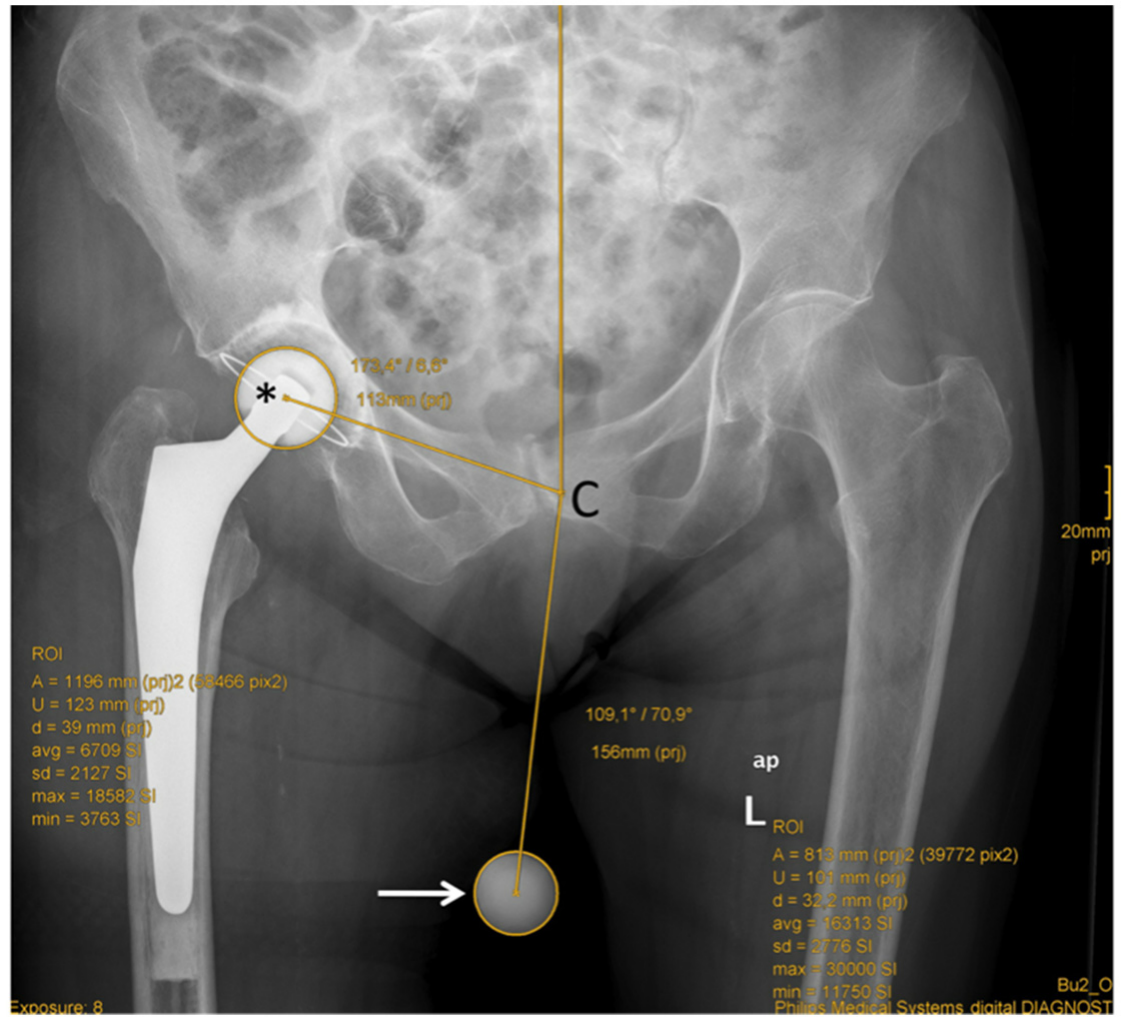
Standing AP radiograph of the pelvis with internal (asterix) and external calibration marker (arrow) and performed measurements: central beam (C); marker diameter; distances of the markers from the central beam; position of the markers.

Analysis of the radiographs was undertaken using a PACS client (IMPAX EE, AGFA HealthCare GmbH, Bonn, Germany). The following measurements were recorded (Fig 3): identification of the center of the image (central beam), the diameters of the ICM and ECM, the position of the markers in degrees (0° at 12 o’clock, clockwise) and the distance of the center of the markers from the central beam in millimeters.

The medical records of each patient were reviewed using ORBIS (AGFA HealthCare GmbH). Each patient’s age, height, and weight were recorded, along with the size and type of implant. The BMI was calculated and patients were grouped according to the World Health Organization definition as underweight (<18.5 kg/m^2^), normal (18.5–29.9 kg/m^2^), or obese (≥30 kg/m^2^).

The magnifications of the ECM and ICM as a percentage were calculated by the formula:

(projected diameter/true diameter) *100 (5)

Two independent and blinded observers analyzed all radiographs (LR, JB). Repeated measures were performed 3 months after the first analysis by one observer (LR), blinded to the previous results.

#### Statistics

For descriptive analysis, absolute mean values and ranges of the measured variables are reported. Variables were tested for normality using the Kolmogorov–Smirnov test. Exploratory analysis was performed using the unpaired two-sided *t-*test for normally distributed independent variables (i.e., object distances from central beam). Intraclass correlation with a 95% confidence interval for the assessment of ECM or ICM diameter, position, distance, and magnification was calculated for the repeated measures of one observer and for two independent observers. Pearson correlation coefficients (r) were used to assess the relationship between magnification, position, and distance of ICM and ECM, and height, weight, BMI, and BMI group. Results with p values <0.05 were considered statistically significant. IBM SPSS Statistics for Macintosh version 22.0 (IBM Corporation, Armonk, NY, USA) or Microsoft Excel 2008 for Mac version 12.3.6 (Microsoft Corporation, Redmond, WA, USA) were used for all analyses.

### Ethics

The study protocol followed the principles of the Declaration of Helsinki and was approved by the local ethic committee of the University Hospital of Cologne. Correspondence number 14–158. For this retrospective study, no consent was neccessary. All data were documented and analyzed anonymized and de-identified.

## Results

### Theoretical magnification of spheres caused by vertical and horizontal displacement

**Fig 4:**
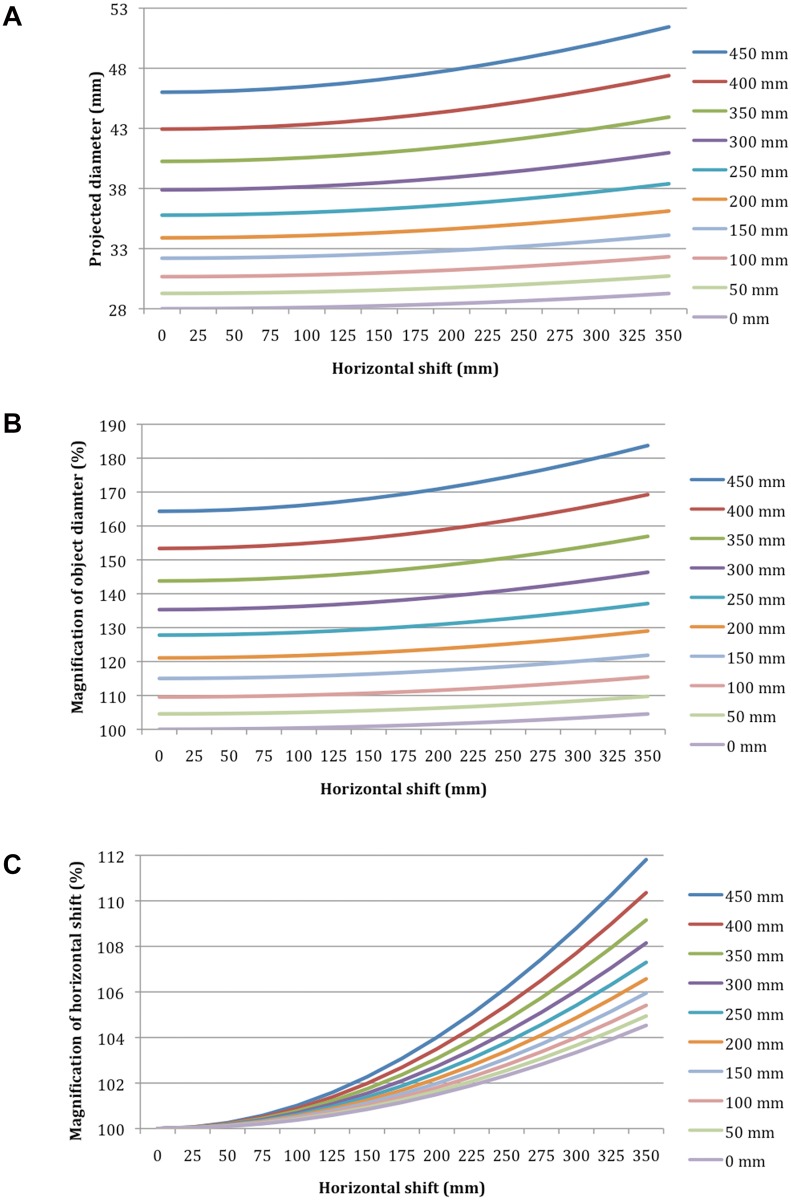
Graphs for radiographic magnification. (A) Absolute size of the projection of a 28 mm sphere; (B) percentage of the overall magnification for a sphere of any diameter; (C) magnification of the horizontal shift alone. Each line represents a vertical position between 0 and 450 mm above the detector in 50 mm increments.

The calculated absolute size of projection of a 28-mm sphere and extent of magnification are shown in Fig 4A–4C. A table showing the results for 50-mm increments in the vertical (0–450 mm) and horizontal (0–350 mm) axes may be viewed in the supplementary data (S2 Table).

### Projected diameter and position of external and internal calibration markers

Repeated observations are shown as the mean and range in Table 1. Three ECMs were positioned adjacent to the left lateral thigh, five to the right lateral thigh, and 92 between the legs. Sixty ICMs were on the left. Subgroup-specific means of repeated measurements are shown in Table 2.

**Table 1:** Baseline parameters of radiographic analysis.

	Measurement 1*		Measurement 2*		Measurement 3**	
	Mean	Range	Mean	Range	Mean	Range
ECM diameter	35.1	31–43	35.3	31.4–43	35.2	30.6–41.5
ICM diameter	37.7	26.8–44.7	37.9	26.9–44.5	37.8	27.0–44.8
ECM position	182.9	104.2–290.7	183.0	104.1–290.0	183.6	104.5–290.2
ICM position	156.0	34.5–323.90	156.0	34.1–324.0	156.4	35.0–330.4
ECM distance	142.3	35.0–245.0	142.4	34.7–246.0	142.8	34.0–244.0
ICM distance	127.5	86.1–179.0	127.6	86.6–179.0	127.5	86.1–180.0
ECM distance calibrated	113.9	26.3–211.8	113.3	25.8–205.9	114.2	25.6–213.4
ICM distance calibrated	104.2	73.5–144.9	103.8	73.1–145.3	104.5	70.4–145.7

Abbreviations: external calibration marker (ECM), internal calibration marker (ICM).

* Observer 1.

** Observer 2.

**Table 2:** Subgroups of external and internal calibration markers.

	N	Means of all measurements*	
		Mean	Range
ECM position			
- left thigh	3	120.7	104.3–133.4
- right thigh	5	258.0	229.7–290.9
- between legs	92	181.1	146.0–212.8
ICM position			
- left hip	60	58.4	34.5–95.7
- right hip	40	302.8	267.1–324.0
ECM distance			
- left thigh	3	223.1	188.0–245.0
- right thigh	5	174.5	114.3–219.0
- between leg	92	138.1	34.6–202.3
ICM distance			
- left hip	60	125.0	93.1–179.3
- right hip	40	131.3	86.3–173.7

Subgroups of external and internal calibration markers regarding position in degree and distance from the centre of the X-ray beam.

* Means of repeated measurements were calculated for each case. Abbreviations: external calibration marker (ECM), internal calibration marker (ICM).

### Magnification of external and internal calibration markers

**Fig 5:**
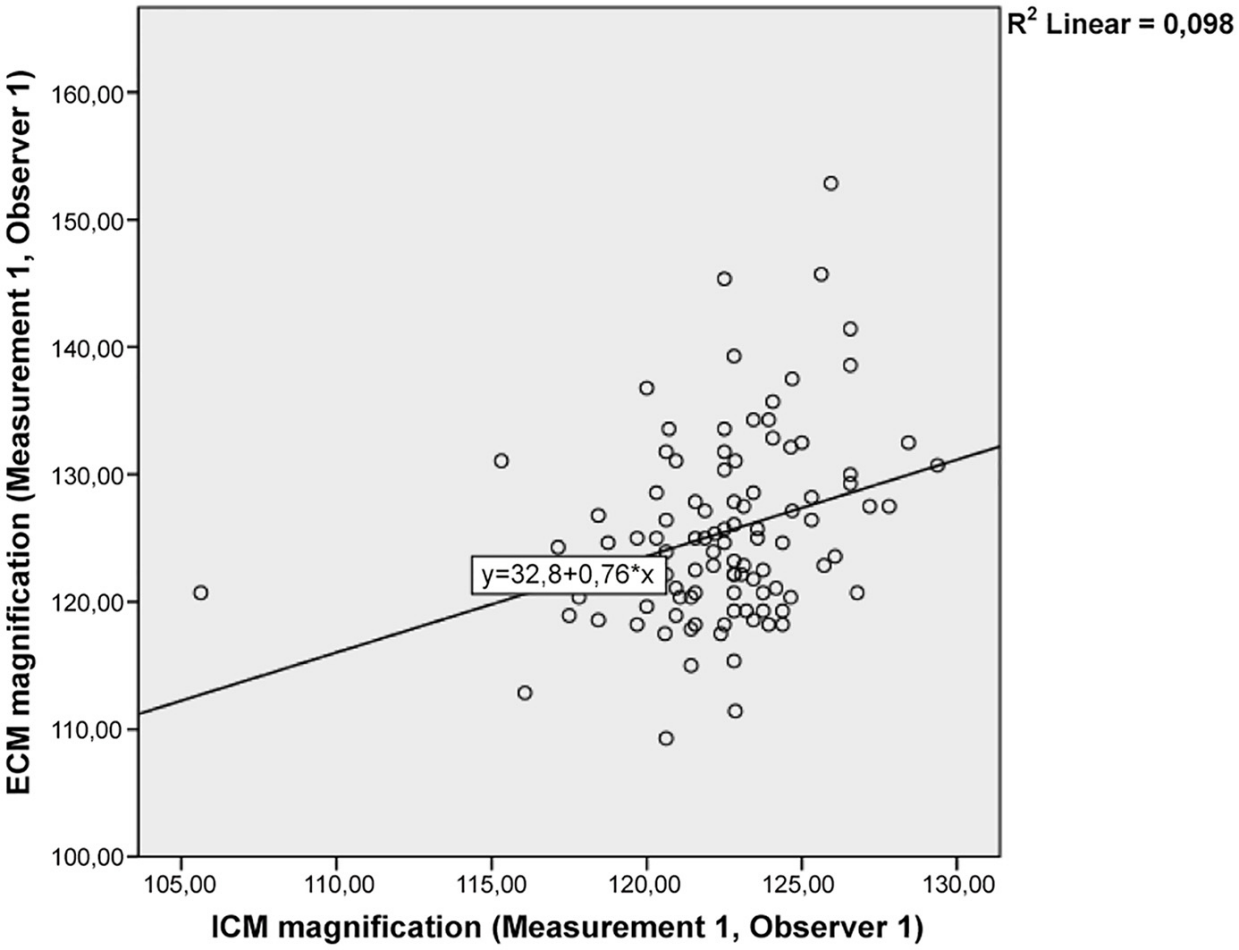
Scatter-plot of the correlation of internal (ICM) and external calibration marker (ECM) of one measurement with the corresponding R2. Linear regression line included.

The magnification of ECMs, ICMs, and the proportional difference are shown in Table 3. The cumulated mean difference between measurements was 3.0% (standard deviation 7.3%). There was a weak correlation between the extent of magnification of the ICM and that of the ECM (Table 4 and Fig 5).

**Table 3:** Magnification of internal and external calibration markers and difference of each case in percent.

	Measurement 1*		Measurement 2*		Measurement 3**	
	Mean	Range	Mean	Range	Mean	Range
ECM	125.5	109.3–152.9	126.1	112.1–153.6	125.7	109.3–148.2
ICM	122.5	105.6–129.4	123.0	105.9–130.6	122.6	105.6–132.2
Difference	3.0	-11.4–26.9	3.0	-11.3–26.4	3.1	-11.8–21.8

Abbreviations: external calibration marker (ECM), internal calibration marker (ICM).

* Observer 1.

** Observer 2.

**Table 4:** Spearman correlation coefficients (r) for magnification of internal and external calibration markers and repeated measurements.

		ICM		
		Measurement 1*	Measurement 2*	Measurement 3**
ECM	Measurement 1*	0.321*	0.355*	0.332*
	Measurement 2*	0.320*	0.361*	0.340*
	Measurement 3**	0.297*	0.344*	0.315*

Abbreviations: external calibration marker (ECM), internal calibration marker (ICM). * Two-sided p < 0.01.

* Observer 1.

** Observer 2.

### Intraclass correlation coefficients for repeated measures

The ICC for inter- and intra-observer reliability of the measured parameters and of the calculated magnification factors of the ECM and ICM are given in Table 5.

**Table 5:** Intraclass correlation coefficients for intra- and inter-observer reliability.

	Intraobserver ICC			Interobserver ICC		
	Mean	CI	p	Mean	CI	p
ECM diameter	0.983	0.976–0.989	0.000	0.971	0.957–0.980	0.000
ICM diameter	0.988	0.982–0.992	0.000	0.991	0.987–0.994	0.000
ECM position	1.000	1.000–1.000	0.000	0.995	0.993–0.997	0.000
ICM position	1.000	1.000–1.000	0.000	1.000	1.000–1.000	0.000
ECM distance	1.000	1.000–1.000	0.000	0.999	0.999–1.000	0.000
ICM distance	0.999	0.999–1.000	0.000	0.998	0.996–0.998	0.000
ECM magnification	0.983	0.976–0.989	0.000	0.971	0.957–0.980	0.000
ICM magnification	0.882	0.830–0.919	0.000	0.920	0.884–0.945	0.000

Mean, 95% confidence-interval (CI) and level of significance (p) are given.

### Position of ECMs and influence on the precision of magnification

Markers located laterally to the thigh and those between the legs were significantly more distant from the center of the X-ray beam (p = 0.006). Detailed information on the evaluation of the position of the ICM and ECM is given in Table 2. The mean calibrated distance of the ECM positioned between the legs was 110.0 (25.9–169.8) mm, for the ICM it was 104.0 (72.5–145.3) mm (p = 0.003).

### Influence of height, weight, and BMI on the precision of external calibration markers

Data were available for 99 patients. Mean height, weight, and BMI were 1.68 (1.40–1.90) m, 73.8 (40–130) kg, and 26.2 (13.4–44.5) kg/m^2^, respectively; 79 patients had normal BMI, 18 were obese, and two were underweight.

Height did not correlate with the magnification of either ECM (r = 0.088, p = 0.385) or ICM (r = 0.095, p = 0.352). There were weak correlations between ECM and ICM and weight (ECM: r = 0.469, p = 0.000; ICM: r = 0.457, p = 0.000), BMI (ECM: r = 0.428, p = 0.000; ICM: r = 0.420, p = 0.000), and BMI group (ECM: r = 0.328, p = 0.001; ICM: r = 0.287, p = 0.004).

## Discussion

Preoperative templating is the accepted standard for planning total hip arthroplasty, and digital techniques have almost completely superseded the use of acetate templating [1]. In digital radiography, calibration is inevitably needed to inform the correct choice of implant size [2,4]. Several investigators have examined the influence of different calibration techniques, including ECMs positioned at the lateral thigh or between the legs [3], radio-opaque discs on the X-ray table [2,7], or other fixed calibration methods [6]. However, most focused exclusively on the vertical position of markers and the hip in the X-ray beam, and therefore did not assess the influence of horizontal shifting of objects in the projected beam. Only The *et al*. have examined this effect in theory and experimentally[5]. Notably, most authors have assumed the projection of a sphere to be identical to that of a horizontal disc of the same diameter, and have not fully taken into account the underlying geometrical principles [4,5].

The effect of horizontal shifting is small compared with that of vertical shifting, but the magnification may reach 1–2% with a horizontal displacement of 100–175 mm depending on the vertical height (Fig 4c). In our cohort, the mean calibrated horizontal shifting of the ECM was 114 (26–210) mm and 104 (73–145) mm for the ICM. Thus, the magnification effect of a horizontal shift is about 1% (0.5–4.0%) for an object 250 mm from the detector, resulting in an error in the assumption of vertical shift of 15–20 mm. Still, the *in vivo* effect is smaller, given that both ECM and ICM are horizontally shifted and only the difference is important. Nonetheless, larger differences should be avoided to prevent clinically meaningful influences on magnification.

In the radiographic analysis, excellent reliability was found for all parameters, underlining the precision of digital measurements and highlighting the observed differences between ECM and ICM.

The position of the ECM relative to the patient is important in the identification of anatomical landmarks at the correct vertical height [2]. While lateral thigh positioning allows better identification of the greater trochanter, this technique is not a reliable means of identifying the height of the hip, and the horizontal shift is by definition larger than that of the hip [3]. Bayne and colleagues also reported a magnification error of 8.86% between the ECM and ICM[3]. In our study, the small number of laterally positioned ECMs makes it difficult to draw any firm conclusions from the analysis, but previously lateral positioning has been shown to be less reliable than other positions [2]. The absolute difference of magnification in this study was 3.1% (0–21.8%). Other investigators have reported the ECM error to be 1.2% and 6.8% [6]. Besides positioning, there is considerable controversy regarding the form and type of the ECM [2,6–8]. Simpler, but potentially more reliable, markers are discs or coins that can be placed on the X-ray table, leading to a fixed magnification in all radiographs and eliminating positional errors [7,9]. Various authors found a fixed magnification based on a retrospective analysis of an ICM to be the most reliable method of templating in their cohorts [2]. Considering the high variability of ECM positioning and the low correlation between ICM and ECM magnification (Fig 5), this is a logical conclusion to make. However, using fixed magnification might be less precise in obese patients or those with anatomical characteristics that vary widely from the mean of the population used to define the fixed magnification [10].

The influence of body weight and BMI on magnification is still not fully understood. Weight has been identified as a factor affecting magnification, while BMI has not [10]. This finding was not supported by our data: weight only correlated weakly with ICM and ECM magnification. Regardless, the vertical height depends on the volume of soft tissue between the ROI and the detector or table. This distance can be estimated by computed tomography [8]; however, the computed tomography table is curved and pelvic radiographs may have been taken either standing or lying on a flat table with a soft pad under the patient, leading to differences in height between computed tomography and plain radiographs.

Our study has some limitations. First, all radiographs were taken in the standing position, so the distance from the detector was not necessarily as small as possible. While there may be less magnification in the supine position, the underlying principles of this study do not depend on the position of the patient. Second, the height, weight, and BMI were acquired retrospectively from the patients’ records. Prospective data acquisition might be a more reliable means of recording biometric characteristics more accurately and precisely.

In conclusion, we derived formulae for the projection of spheres relative to their position in the X-ray beam. We found excellent intra- and inter-observer reliability of the radiographic measurements and magnification of internal and external calibration markers. However, the correlation between external and internal calibration markers was poor, indicating the need for a better calibration method. Weight and BMI were weakly correlated with the magnification of both types of marker; future studies should examine these relationships in more detail.

## Supporting Information

S1 Table Repeated measurements of internal calibration marker (ICM). (DOCX)

S2 Table Absolute magnification of a 28 mm diameter sphere in mm. (DOCX)

S3 Table Repeated measurements of external calibration marker (ECM). (DOCX)

S1 Text Derivation of formula 4. (DOCX)

S2 Text Dereviation of the used formulae as PDF file. (PDF)

S3 Text Dereviation of the used formulae as TEX file. (TEX)
