# Supplementary material for: Calibration Markers for Digital Templating in Total Hip Arthroplasty
Source: PLoS One. 2015 Jul 13;10(7):e0128529. doi: 10.1371/journal.pone.0128529 (PMC4500467; doi:10.1371/journal.pone.0128529)
Supplement: S1 Table — (DOCX) [file pone.0128529.s001.docx]

**S1 Table: Repeated measurements of internal calibration marker (ICM).** Abbreviations: true diameter of ICM (ICM_T_); projected diamter of ICM (ICM_P_). Distance = distance from central beam in mm. Position = clockwise location of marker in degree.

|  |  | **Observer 1, measurement 1** | | | **Observer 1, measurement 1** | | | **Observer 2** | | |
| --- | --- | --- | --- | --- | --- | --- | --- | --- | --- | --- |
| **StudyID** | **ICM_T_** | **ICM_P_** | **Position** | **Distance** | **ICM_P_** | **Position** | **Distance** | **ICM_P_** | **Position** | **Distance** |
| 1 | 32,0 | 40,2 | 74,0 | 111,0 | 40,2 | 73,9 | 110,0 | 39,4 | 73,4 | 112,0 |
| 2 | 28,0 | 34,2 | 48,1 | 145,0 | 34,6 | 48,2 | 145,0 | 33,9 | 47,0 | 145,0 |
| 3 | 32,0 | 37,9 | 291,4 | 97,8 | 37,4 | 292,0 | 99,2 | 37,7 | 290,3 | 98,7 |
| 4 | 32,0 | 39,6 | 267,5 | 109,0 | 39,2 | 267,2 | 109,0 | 39,2 | 266,6 | 110,0 |
| 5 | 32,0 | 40,5 | 277,4 | 111,0 | 40,3 | 277,9 | 112,0 | 40,1 | 275,1 | 111,0 |
| 6 | 32,0 | 38,7 | 55,5 | 122,0 | 38,6 | 55,5 | 121,0 | 39,3 | 55,0 | 121,0 |
| 7 | 32,0 | 38,9 | 292,2 | 121,0 | 39,5 | 292,0 | 122,0 | 39,1 | 292,8 | 123,0 |
| 8 | 32,0 | 38,7 | 269,8 | 133,0 | 38,8 | 269,5 | 133,0 | 38,5 | 268,0 | 131,0 |
| 9 | 32,0 | 39,3 | 60,7 | 122,0 | 39,0 | 60,2 | 122,0 | 39,0 | 58,7 | 120,0 |
| 10 | 32,0 | 38,6 | 95,4 | 120,0 | 38,8 | 95,5 | 119,0 | 38,7 | 96,2 | 120,0 |
| 11 | 32,0 | 41,4 | 66,0 | 132,0 | 40,9 | 66,1 | 132,0 | 40,4 | 66,3 | 133,0 |
| 12 | 28,0 | 32,8 | 286,6 | 86,1 | 33,1 | 286,0 | 86,6 | 33,3 | 289,1 | 86,1 |
| 13 | 32,0 | 38,4 | 50,0 | 129,0 | 38,2 | 49,8 | 129,0 | 38,2 | 50,4 | 130,0 |
| 14 | 32,0 | 39,7 | 84,1 | 103,0 | 40,3 | 84,9 | 102,0 | 40,3 | 84,2 | 101,0 |
| 15 | 32,0 | 39,0 | 308,7 | 134,0 | 39,3 | 308,7 | 135,0 | 39,3 | 307,7 | 134,0 |
| 16 | 32,0 | 39,5 | 48,2 | 145,0 | 39,3 | 48,3 | 145,0 | 39,2 | 48,8 | 145,0 |
| 17 | 32,0 | 39,6 | 70,0 | 99,3 | 39,5 | 70,4 | 99,1 | 38,9 | 70,7 | 99,0 |
| 18 | 32,0 | 39,2 | 291,0 | 104,0 | 39,2 | 291,4 | 104,0 | 39,2 | 291,0 | 104,0 |
| 19 | 32,0 | 33,8 | 41,4 | 125,0 | 33,9 | 41,4 | 125,0 | 33,8 | 41,4 | 125,0 |
| 20 | 32,0 | 40,9 | 299,5 | 123,0 | 40,8 | 299,3 | 123,0 | 40,7 | 298,5 | 123,0 |
| 21 | 32,0 | 40,7 | 314,0 | 174,0 | 41,0 | 313,8 | 173,0 | 40,7 | 317,0 | 174,0 |
| 22 | 32,0 | 39,0 | 83,4 | 107,0 | 38,8 | 83,5 | 107,0 | 39,0 | 84,4 | 108,0 |
| 23 | 32,0 | 38,9 | 299,6 | 112,0 | 39,0 | 299,5 | 113,0 | 39,3 | 302,9 | 111,0 |
| 24 | 28,0 | 34,6 | 310,5 | 133,0 | 34,6 | 310,1 | 133,0 | 34,6 | 310,5 | 133,0 |
| 25 | 32,0 | 40,0 | 69,1 | 110,0 | 40,5 | 68,7 | 111,0 | 40,0 | 69,1 | 110,0 |
| 26 | 32,0 | 39,3 | 59,2 | 124,0 | 39,6 | 59,3 | 124,0 | 39,3 | 59,2 | 124,0 |
| 27 | 32,0 | 39,3 | 35,7 | 146,0 | 39,5 | 35,7 | 145,0 | 39,3 | 35,7 | 146,0 |
| 28 | 32,0 | 40,5 | 40,0 | 156,0 | 40,6 | 39,9 | 156,0 | 40,0 | 40,5 | 157,0 |
| 29 | 32,0 | 38,6 | 56,8 | 97,5 | 38,2 | 56,2 | 97,1 | 38,6 | 56,8 | 98,0 |
| 30 | 28,0 | 34,6 | 43,6 | 179,0 | 34,5 | 43,6 | 179,0 | 34,6 | 44,6 | 180,0 |
| 31 | 32,0 | 40,1 | 314,9 | 156,0 | 40,1 | 314,9 | 156,0 | 40,4 | 312,5 | 155,0 |
| 32 | 32,0 | 39,4 | 90,5 | 93,0 | 38,9 | 90,7 | 93,9 | 40,3 | 92,0 | 94,0 |
| 33 | 32,0 | 38,5 | 296,7 | 122,0 | 38,5 | 296,8 | 121,0 | 38,5 | 296,1 | 123,0 |
| 34 | 32,0 | 39,3 | 59,0 | 133,0 | 39,5 | 59,1 | 134,0 | 39,5 | 57,9 | 132,0 |
| 35 | 32,0 | 39,7 | 313,9 | 157,0 | 39,0 | 314,3 | 157,0 | 39,9 | 315,9 | 157,0 |
| 36 | 32,0 | 38,7 | 323,0 | 160,0 | 39,0 | 322,9 | 160,0 | 39,0 | 325,0 | 160,0 |
| 37 | 28,0 | 34,4 | 68,0 | 111,0 | 35,0 | 67,9 | 111,0 | 34,6 | 69,0 | 110,0 |
| 38 | 32,0 | 39,8 | 284,4 | 126,0 | 39,3 | 284,6 | 127,0 | 39,2 | 285,5 | 125,0 |
| 39 | 32,0 | 39,8 | 68,2 | 141,0 | 40,1 | 68,6 | 142,0 | 39,5 | 69,2 | 141,0 |
| 40 | 28,0 | 34,9 | 304,2 | 119,0 | 35,1 | 304,7 | 119,0 | 35,0 | 306,2 | 120,0 |
| 41 | 32,0 | 39,5 | 54,1 | 144,0 | 39,8 | 53,7 | 144,0 | 40,0 | 53,2 | 144,0 |
| 42 | 28,0 | 34,0 | 50,2 | 126,0 | 35,1 | 50,0 | 126,0 | 34,5 | 50,0 | 126,0 |
| 43 | 28,0 | 34,3 | 78,7 | 94,2 | 34,6 | 78,4 | 95,0 | 33,8 | 79,3 | 95,0 |
| 44 | 32,0 | 39,8 | 314,8 | 137,0 | 40,1 | 314,6 | 136,0 | 40,1 | 315,5 | 137,0 |
| 45 | 28,0 | 34,2 | 304,3 | 127,0 | 34,4 | 304,5 | 128,0 | 34,5 | 303,5 | 128,0 |
| 46 | 32,0 | 39,1 | 322,1 | 161,0 | 39,3 | 322,2 | 161,0 | 38,9 | 322,1 | 160,0 |
| 47 | 32,0 | 39,3 | 86,9 | 104,0 | 39,4 | 86,8 | 104,0 | 40,1 | 88,7 | 106,0 |
| 48 | 32,0 | 39,4 | 57,7 | 104,0 | 39,8 | 58,1 | 104,0 | 39,1 | 56,7 | 105,0 |
| 49 | 28,0 | 35,3 | 58,0 | 121,0 | 35,2 | 57,9 | 121,0 | 35,3 | 60,0 | 123,0 |
| 50 | 28,0 | 34,3 | 301,2 | 130,0 | 34,0 | 301,2 | 130,0 | 34,5 | 301,4 | 130,0 |
| 51 | 32,0 | 39,2 | 68,3 | 122,0 | 39,7 | 67,6 | 122,0 | 38,9 | 68,3 | 119,0 |
| 52 | 32,0 | 39,3 | 57,4 | 118,0 | 39,3 | 57,1 | 117,0 | 39,9 | 59,4 | 118,0 |
| 53 | 28,0 | 34,9 | 303,9 | 130,0 | 34,8 | 303,8 | 130,0 | 35,5 | 305,9 | 130,0 |
| 54 | 32,0 | 37,9 | 67,3 | 107,0 | 38,2 | 67,1 | 106,0 | 37,2 | 65,6 | 110,0 |
| 55 | 32,0 | 39,5 | 63,6 | 111,0 | 40,5 | 63,1 | 110,0 | 39,5 | 66,5 | 111,0 |
| 56 | 32,0 | 40,5 | 34,5 | 151,0 | 40,4 | 34,1 | 151,0 | 40,7 | 35,0 | 149,0 |
| 57 | 28,0 | 34,3 | 58,8 | 134,0 | 34,7 | 58,5 | 134,0 | 34,3 | 59,8 | 133,0 |
| 58 | 32,0 | 40,3 | 81,7 | 93,2 | 40,7 | 81,4 | 93,0 | 42,3 | 82,5 | 93,0 |
| 59 | 32,0 | 39,9 | 282,2 | 114,0 | 40,3 | 286,2 | 114,0 | 40,2 | 282,6 | 114,0 |
| 60 | 32,0 | 38,5 | 49,9 | 124,0 | 38,8 | 49,8 | 125,0 | 38,5 | 52,0 | 126,0 |
| 61 | 32,0 | 40,1 | 49,7 | 165,0 | 40,0 | 49,5 | 165,0 | 40,3 | 50,1 | 166,0 |
| 62 | 32,0 | 38,6 | 49,8 | 140,0 | 38,9 | 49,5 | 140,0 | 38,3 | 46,9 | 138,0 |
| 63 | 32,0 | 39,3 | 58,7 | 111,0 | 39,3 | 59,0 | 111,0 | 39,2 | 58,9 | 111,0 |
| 64 | 32,0 | 39,2 | 323,9 | 169,0 | 39,1 | 324,0 | 170,0 | 39,2 | 324,2 | 167,0 |
| 65 | 28,0 | 34,4 | 59,4 | 121,0 | 34,7 | 59,3 | 122,0 | 34,4 | 59,9 | 122,0 |
| 66 | 22,2 | 27,2 | 298,5 | 123,0 | 27,1 | 299,0 | 125,0 | 27,0 | 299,1 | 125,0 |
| 67 | 32,0 | 39,5 | 295,4 | 105,0 | 39,7 | 295,8 | 105,0 | 39,5 | 297,4 | 105,0 |
| 68 | 28,0 | 35,5 | 52,9 | 138,0 | 35,5 | 52,8 | 139,0 | 35,8 | 52,2 | 138,0 |
| 69 | 28,0 | 34,0 | 51,1 | 111,0 | 33,8 | 51,0 | 111,0 | 34,0 | 51,3 | 112,0 |
| 70 | 28,0 | 33,9 | 40,3 | 122,0 | 34,1 | 40,3 | 122,0 | 33,9 | 40,8 | 123,0 |
| 71 | 32,0 | 40,5 | 54,6 | 159,0 | 40,6 | 54,5 | 159,0 | 40,4 | 54,8 | 161,0 |
| 72 | 22,2 | 26,8 | 41,4 | 122,0 | 26,9 | 41,2 | 123,0 | 27,1 | 43,0 | 120,0 |
| 73 | 28,0 | 33,8 | 70,7 | 106,0 | 34,1 | 70,4 | 106,0 | 33,2 | 70,9 | 103,0 |
| 74 | 36,0 | 44,3 | 315,6 | 162,0 | 44,5 | 315,9 | 163,0 | 44,8 | 318,8 | 165,0 |
| 75 | 28,0 | 34,7 | 55,6 | 105,0 | 34,6 | 55,5 | 104,0 | 34,7 | 55,1 | 105,0 |
| 76 | 28,0 | 34,7 | 309,3 | 145,0 | 35,3 | 308,9 | 144,0 | 34,4 | 312,5 | 144,0 |
| 77 | 28,0 | 32,5 | 59,0 | 142,0 | 32,6 | 59,2 | 142,0 | 32,5 | 59,4 | 144,0 |
| 78 | 28,0 | 33,6 | 58,6 | 117,0 | 34,0 | 58,3 | 117,0 | 33,6 | 57,6 | 117,0 |
| 79 | 28,0 | 34,3 | 87,0 | 104,0 | 34,9 | 86,6 | 104,0 | 34,0 | 89,3 | 102,0 |
| 80 | 32,0 | 39,3 | 321,6 | 152,0 | 38,8 | 322,0 | 151,0 | 39,3 | 326,1 | 150,0 |
| 81 | 28,0 | 33,6 | 45,4 | 135,0 | 34,4 | 44,9 | 135,0 | 33,6 | 35,9 | 133,0 |
| 82 | 28,0 | 34,0 | 285,9 | 99,2 | 33,2 | 284,7 | 99,0 | 33,6 | 288,0 | 97,0 |
| 83 | 28,0 | 34,5 | 51,1 | 115,0 | 34,1 | 51,2 | 114,0 | 34,5 | 50,1 | 114,0 |
| 84 | 32,0 | 38,3 | 314,1 | 149,0 | 38,9 | 313,9 | 149,0 | 38,5 | 316,1 | 145,0 |
| 85 | 32,0 | 38,9 | 314,2 | 132,0 | 39,6 | 313,9 | 131,0 | 39,0 | 314,0 | 135,0 |
| 86 | 36,0 | 44,7 | 306,9 | 135,0 | 43,8 | 306,0 | 136,0 | 44,2 | 306,7 | 134,0 |
| 87 | 32,0 | 37,7 | 48,0 | 118,0 | 37,8 | 47,8 | 118,0 | 37,9 | 48,6 | 119,0 |
| 88 | 32,0 | 38,3 | 44,3 | 151,0 | 39,2 | 44,6 | 151,0 | 38,3 | 44,7 | 150,0 |
| 89 | 32,0 | 39,9 | 314,4 | 152,0 | 41,1 | 313,8 | 152,0 | 40,0 | 312,0 | 152,0 |
| 90 | 32,0 | 39,6 | 286,6 | 109,0 | 40,1 | 287,9 | 108,0 | 39,8 | 288,0 | 111,0 |
| 91 | 32,0 | 37,6 | 35,8 | 165,0 | 39,0 | 35,8 | 164,0 | 37,6 | 38,0 | 166,0 |
| 92 | 32,0 | 36,9 | 63,1 | 134,0 | 37,3 | 63,3 | 134,0 | 36,9 | 63,1 | 134,0 |
| 93 | 32,0 | 39,3 | 315,2 | 154,0 | 39,5 | 315,1 | 154,0 | 39,7 | 310,3 | 154,0 |
| 94 | 32,0 | 41,1 | 295,6 | 117,0 | 41,8 | 296,7 | 119,0 | 41,0 | 298,8 | 114,0 |
| 95 | 32,0 | 38,6 | 49,3 | 111,0 | 39,5 | 49,7 | 112,0 | 38,6 | 49,3 | 111,0 |
| 96 | 32,0 | 38,9 | 305,8 | 123,0 | 39,4 | 305,3 | 123,0 | 39,2 | 307,8 | 123,0 |
| 97 | 32,0 | 38,0 | 45,0 | 141,0 | 39,2 | 45,0 | 141,0 | 38,4 | 44,2 | 140,0 |
| 98 | 32,0 | 38,9 | 50,3 | 142,0 | 38,5 | 50,7 | 142,0 | 39,3 | 53,1 | 140,0 |
| 99 | 32,0 | 38,6 | 321,1 | 148,0 | 39,5 | 320,5 | 149,0 | 38,3 | 330,4 | 151,0 |
| 100 | 28,0 | 35,2 | 48,7 | 124,0 | 34,7 | 48,4 | 126,0 | 35,0 | 47,1 | 121,0 |
