# Supplementary material for: Calibration Markers for Digital Templating in Total Hip Arthroplasty
Source: PLoS One. 2015 Jul 13;10(7):e0128529. doi: 10.1371/journal.pone.0128529 (PMC4500467; doi:10.1371/journal.pone.0128529)
Supplement: S2 Table — (DOCX) [file pone.0128529.s002.docx]

**S2 Table: Absolute magnification of a 28 mm diameter sphere in mm.**

|  | *x_0_* | | | | | | | | | | | | | | |
| --- | --- | --- | --- | --- | --- | --- | --- | --- | --- | --- | --- | --- | --- | --- | --- |
| *z_0_* | 0 | 25 | 50 | 75 | 100 | 125 | 150 | 175 | 200 | 225 | 250 | 275 | 300 | 325 | 350 |
| 450 | 46,01 | 46,04 | 46,13 | 46,27 | 46,48 | 46,74 | 47,05 | 47,43 | 47,85 | 48,33 | 48,86 | 49,43 | 50,06 | 50,73 | 51,44 |
| 400 | 42,94 | 42,96 | 43,04 | 43,16 | 43,32 | 43,53 | 43,79 | 44,09 | 44,44 | 44,83 | 45,26 | 45,74 | 46,25 | 46,80 | 47,39 |
| 350 | 40,26 | 40,28 | 40,33 | 40,43 | 40,57 | 40,74 | 40,96 | 41,21 | 41,50 | 41,82 | 42,18 | 42,57 | 42,99 | 43,45 | 43,94 |
| 300 | 37,89 | 37,90 | 37,95 | 38,03 | 38,15 | 38,30 | 38,47 | 38,68 | 38,92 | 39,19 | 39,49 | 39,82 | 40,18 | 40,56 | 40,97 |
| 250 | 35,78 | 35,80 | 35,84 | 35,91 | 36,00 | 36,13 | 36,28 | 36,45 | 36,66 | 36,88 | 37,14 | 37,42 | 37,72 | 38,04 | 38,39 |
| 200 | 33,90 | 33,91 | 33,95 | 34,00 | 34,09 | 34,19 | 34,32 | 34,47 | 34,64 | 34,84 | 35,05 | 35,29 | 35,55 | 35,83 | 36,13 |
| 150 | 32,20 | 32,21 | 32,24 | 32,29 | 32,36 | 32,45 | 32,56 | 32,69 | 32,84 | 33,01 | 33,19 | 33,40 | 33,62 | 33,86 | 34,12 |
| 100 | 30,67 | 30,68 | 30,70 | 30,75 | 30,81 | 30,89 | 30,98 | 31,09 | 31,22 | 31,37 | 31,53 | 31,70 | 31,90 | 32,11 | 32,33 |
| 50 | 29,28 | 29,28 | 29,31 | 29,34 | 29,40 | 29,46 | 29,55 | 29,64 | 29,76 | 29,88 | 30,02 | 30,18 | 30,34 | 30,53 | 30,72 |
| 0 | 28,00 | 28,01 | 28,03 | 28,06 | 28,11 | 28,17 | 28,24 | 28,32 | 28,42 | 28,53 | 28,66 | 28,79 | 28,94 | 29,10 | 29,27 |

Absolute magnification. Projected diameter of a 28 mm diameter sphere. Results for 50 mm increments of vertical (0-450 mm) and 25 mm increments horizontal shift (0-350 mm). Focus-detector distance set at 1150 mm. *z_0_* = object-detector distance in mm. *x_0_* = vertical shift from central beam in mm.
