# Supplementary material for: Calibration Markers for Digital Templating in Total Hip Arthroplasty
Source: PLoS One. 2015 Jul 13;10(7):e0128529. doi: 10.1371/journal.pone.0128529 (PMC4500467; doi:10.1371/journal.pone.0128529)
Supplement: S3 Table — (DOCX) [file pone.0128529.s003.docx]

**S3 Table: Repeated measurements of external calibration marker (ECM).** Abbreviations: true diameter of ECM (ECM_T_); projected diamter of ECM (ECM_P_). Distance = distance from central beam in mm. Position = clockwise location of marker in degree.

|  |  | **Observer 1, measurement 1** | | | **Observer 1, measurement 1** | | | **Observer 2** | | |
| --- | --- | --- | --- | --- | --- | --- | --- | --- | --- | --- |
| **StudyID** | **ECM_T_** | **ECM_P_** | **Position** | **Distance** | **ECM_P_** | **Position** | **Distance** | **ECM_P_** | **Position** | **Distance** |
| 1 | 28 | 40,8 | 195,9 | 164,0 | 41,0 | 195,8 | 165,0 | 40,2 | 198 | 164,0 |
| 2 | 28 | 34,7 | 188,5 | 68,3 | 34,8 | 188,0 | 68,5 | 35,0 | 188,2 | 67,7 |
| 3 | 28 | 33,2 | 290,7 | 213,0 | 33,1 | 290,9 | 214,0 | 32,3 | 291,2 | 212,0 |
| 4 | 28 | 34,3 | 181,8 | 188,0 | 34,2 | 181,3 | 189,0 | 34,8 | 182,5 | 188,0 |
| 5 | 28 | 38,8 | 186,1 | 148,0 | 38,7 | 185,7 | 148,0 | 38,9 | 185,2 | 148,0 |
| 6 | 28 | 36,7 | 179,3 | 153,0 | 36,7 | 181,4 | 153,0 | 35,9 | 180,2 | 154,0 |
| 7 | 28 | 35,8 | 179,8 | 104,0 | 35,9 | 180,8 | 105,0 | 36,0 | 180,1 | 105,0 |
| 8 | 28 | 33,9 | 104,2 | 188,0 | 33,5 | 104,1 | 188,0 | 33,5 | 104,5 | 188,0 |
| 9 | 28 | 33,4 | 173,1 | 112,0 | 33,4 | 173,2 | 112,0 | 33,0 | 172,3 | 113,0 |
| 10 | 28 | 35,4 | 188,8 | 159,0 | 35,6 | 188,8 | 160,0 | 35,2 | 188,6 | 160,0 |
| 11 | 28 | 36,6 | 183,1 | 163,0 | 36,9 | 182,9 | 164,0 | 37,2 | 184 | 161,0 |
| 12 | 28 | 34,8 | 183,9 | 178,0 | 34,4 | 183,8 | 177,0 | 34,1 | 184,8 | 178,0 |
| 13 | 28 | 38,3 | 146,4 | 158,0 | 38,6 | 146,9 | 158,0 | 38,9 | 144,8 | 159,0 |
| 14 | 28 | 38,0 | 146,5 | 86,0 | 37,9 | 146,7 | 86,0 | 37,9 | 146,9 | 86,0 |
| 15 | 28 | 35,6 | 200,1 | 59,0 | 35,2 | 200,1 | 58,9 | 35,6 | 201,7 | 59,0 |
| 16 | 28 | 33,2 | 175,9 | 110,0 | 33,4 | 175,9 | 110,0 | 33,3 | 174,8 | 111,0 |
| 17 | 28 | 33,8 | 177,1 | 165,0 | 34,0 | 177,5 | 165,0 | 34,2 | 175,1 | 166,0 |
| 18 | 28 | 33,1 | 168,0 | 142,0 | 33,2 | 167,9 | 141,0 | 33,1 | 168,0 | 142,0 |
| 19 | 28 | 33,8 | 178,9 | 101,0 | 33,9 | 179,2 | 101,0 | 33,8 | 178,9 | 101,0 |
| 20 | 28 | 35,7 | 175,0 | 155,0 | 36,0 | 174,9 | 155,0 | 35,7 | 175,7 | 155,0 |
| 21 | 28 | 35,7 | 166,1 | 122,0 | 35,7 | 166,1 | 123,0 | 35,8 | 165,5 | 121,0 |
| 22 | 28 | 35,0 | 176,9 | 178,0 | 35,2 | 177,0 | 178,0 | 35,2 | 177,7 | 178,0 |
| 23 | 28 | 33,1 | 172,7 | 84,5 | 33,4 | 172,9 | 85,0 | 33,3 | 173,5 | 85,0 |
| 24 | 28 | 35,2 | 181,1 | 129,0 | 35,3 | 180,6 | 129,0 | 35,2 | 181,1 | 129,0 |
| 25 | 28 | 37,1 | 180,8 | 148,0 | 37,4 | 181,0 | 148,0 | 37,1 | 180,8 | 148,0 |
| 26 | 28 | 35,3 | 185,1 | 145,0 | 35,7 | 185,0 | 146,0 | 35,3 | 185,1 | 145,0 |
| 27 | 28 | 35,8 | 183,5 | 175,0 | 36,2 | 183,8 | 175,0 | 35,8 | 183,5 | 175,0 |
| 28 | 28 | 36,4 | 173,1 | 176,0 | 36,7 | 173,9 | 175,0 | 35,0 | 173 | 176,0 |
| 29 | 28 | 34,7 | 178,1 | 176,0 | 34,9 | 178,3 | 176,0 | 34,7 | 178,1 | 176,0 |
| 30 | 28 | 35,0 | 179,9 | 179,0 | 35,3 | 179,6 | 179,0 | 35,0 | 179,9 | 179,0 |
| 31 | 28 | 35,9 | 185,5 | 150,0 | 35,4 | 185,6 | 150,0 | 36,0 | 184,0 | 152,0 |
| 32 | 28 | 35,7 | 184,3 | 142,0 | 36,0 | 184,0 | 143,0 | 35,3 | 182,0 | 144,0 |
| 33 | 28 | 36,0 | 174,4 | 140,0 | 35,7 | 174,1 | 140,0 | 36,1 | 172,4 | 141,0 |
| 34 | 28 | 34,5 | 166,8 | 80,5 | 34,3 | 167,4 | 81,0 | 36,1 | 168,2 | 80,0 |
| 35 | 28 | 37,2 | 203,7 | 35,0 | 37,6 | 204,8 | 34,7 | 37,2 | 205,5 | 34,0 |
| 36 | 28 | 33,3 | 196,4 | 94,5 | 32,9 | 196,5 | 94,2 | 33,8 | 194,4 | 96,0 |
| 37 | 28 | 36,7 | 160,0 | 83,5 | 37,1 | 161,3 | 83,2 | 36,7 | 159,4 | 83,0 |
| 38 | 28 | 33,4 | 183,7 | 129,0 | 33,6 | 184,2 | 129,0 | 33,3 | 181,7 | 129,0 |
| 39 | 28 | 33,1 | 179,4 | 143,0 | 33,1 | 179,4 | 143,0 | 33,1 | 178,4 | 141,0 |
| 40 | 28 | 33,7 | 159,3 | 85,4 | 33,7 | 158,1 | 84,8 | 33,5 | 163,3 | 87,0 |
| 41 | 28 | 34,1 | 188,1 | 123,0 | 34,2 | 188,3 | 122,0 | 34,4 | 186,0 | 122,0 |
| 42 | 28 | 33,7 | 174,1 | 88,9 | 33,7 | 174,9 | 88,5 | 33,7 | 176,1 | 89,0 |
| 43 | 28 | 37,4 | 133,6 | 245,0 | 37,6 | 133,3 | 246,0 | 38,1 | 133,2 | 244,0 |
| 44 | 28 | 34,9 | 181,7 | 156,0 | 34,7 | 181,4 | 156,0 | 35,0 | 180,5 | 155,0 |
| 45 | 28 | 34,4 | 177,8 | 170,0 | 34,7 | 178,0 | 169,0 | 34,2 | 179,1 | 171,0 |
| 46 | 28 | 35,1 | 189,3 | 193,0 | 34,9 | 189,4 | 193,0 | 35,7 | 191,3 | 194,0 |
| 47 | 28 | 39,0 | 211,7 | 193,0 | 39,1 | 211,9 | 193,0 | 39,3 | 214,7 | 195,0 |
| 48 | 28 | 34,4 | 182,3 | 201,0 | 34,1 | 182,3 | 202,0 | 34,4 | 184,3 | 204,0 |
| 49 | 28 | 34,6 | 180,8 | 98,1 | 34,7 | 180,6 | 98,0 | 34,9 | 178,6 | 100,0 |
| 50 | 28 | 36,9 | 188,8 | 152,0 | 36,9 | 188,7 | 151,0 | 35,3 | 188,6 | 150,0 |
| 51 | 28 | 34,9 | 178,4 | 140,0 | 35,2 | 179,0 | 139,0 | 34,9 | 180,2 | 141,0 |
| 52 | 28 | 32,3 | 184,8 | 153,0 | 32,8 | 185,7 | 152,0 | 32,3 | 185,5 | 155,0 |
| 53 | 28 | 37,0 | 189,2 | 132,0 | 37,4 | 189,5 | 131,0 | 37,0 | 192,2 | 132,0 |
| 54 | 28 | 35,5 | 165,1 | 151,0 | 35,8 | 165,5 | 151,0 | 36,7 | 168,5 | 152,0 |
| 55 | 28 | 36,0 | 170,7 | 158,0 | 35,4 | 171,2 | 158,0 | 35,6 | 170,7 | 159,0 |
| 56 | 28 | 36,2 | 179,7 | 166,0 | 35,9 | 180,0 | 166,0 | 36,5 | 181,6 | 168,0 |
| 57 | 28 | 36,5 | 179,4 | 122,0 | 36,3 | 179,7 | 121,0 | 36,5 | 177,6 | 122,0 |
| 58 | 28 | 42,8 | 228,0 | 115,0 | 43,0 | 228,0 | 116,0 | 41,5 | 233,0 | 112,0 |
| 59 | 28 | 38,5 | 186,8 | 181,0 | 38,5 | 186,7 | 181,0 | 38,5 | 185,1 | 182,0 |
| 60 | 28 | 35,0 | 199,0 | 113,0 | 35,4 | 198,7 | 113,0 | 35,0 | 203,3 | 112,0 |
| 61 | 28 | 35,4 | 198,7 | 54,3 | 35,7 | 198,7 | 54,5 | 35,3 | 198,0 | 54,0 |
| 62 | 28 | 36,9 | 187,6 | 170,0 | 37,0 | 187,0 | 169,0 | 37,0 | 190,9 | 171,0 |
| 63 | 28 | 33,8 | 174,9 | 118,0 | 33,5 | 175,1 | 118,0 | 33,5 | 175,3 | 120,0 |
| 64 | 28 | 40,7 | 208,6 | 136,0 | 40,7 | 208,9 | 137,0 | 40,4 | 206,6 | 135,0 |
| 65 | 28 | 31,2 | 124,5 | 236,0 | 32,1 | 124,4 | 236,0 | 31,1 | 124,5 | 237,0 |
| 66 | 28 | 32,9 | 198,0 | 138,0 | 33,0 | 198,8 | 138,0 | 32,5 | 198,1 | 138,0 |
| 67 | 28 | 37,6 | 181,8 | 116,0 | 38,3 | 181,6 | 115,0 | 37,6 | 182,4 | 115,0 |
| 68 | 28 | 33,8 | 164,6 | 83,5 | 34,1 | 163,6 | 84,2 | 33,8 | 166,6 | 85,0 |
| 69 | 28 | 32,2 | 184,8 | 95,9 | 32,7 | 184,8 | 95,6 | 32,1 | 183,9 | 95,0 |
| 70 | 28 | 33,7 | 182,6 | 60,7 | 33,8 | 182,9 | 61,6 | 33,7 | 181,6 | 63,0 |
| 71 | 28 | 39,6 | 178,4 | 173,0 | 39,2 | 178,4 | 174,0 | 40,0 | 178,6 | 177,0 |
| 72 | 28 | 32,9 | 197,9 | 99,2 | 33,3 | 197,9 | 99,1 | 33,4 | 197,9 | 100,0 |
| 73 | 28 | 37,4 | 244,8 | 119,0 | 37,7 | 245,1 | 119,0 | 37,5 | 243,2 | 115,0 |
| 74 | 28 | 34,2 | 175,4 | 200,0 | 34,6 | 175,5 | 200,0 | 34,7 | 176,7 | 204,0 |
| 75 | 28 | 33,1 | 192,8 | 157,0 | 33,0 | 193,0 | 157,0 | 33,1 | 194,0 | 159,0 |
| 76 | 28 | 37,6 | 174,1 | 125,0 | 37,6 | 174,5 | 127,0 | 37,6 | 177,7 | 125,0 |
| 77 | 28 | 31,6 | 269,8 | 208,0 | 31,7 | 270,0 | 206,0 | 32,1 | 272,8 | 211,0 |
| 78 | 28 | 33,5 | 252,8 | 218,0 | 33,7 | 253,2 | 219,0 | 35,4 | 256,3 | 220,0 |
| 79 | 28 | 35,2 | 182,9 | 173,0 | 35,0 | 183,0 | 172,0 | 35,5 | 186,9 | 177,0 |
| 80 | 28 | 34,2 | 175,2 | 152,0 | 34,6 | 174,7 | 152,0 | 34,8 | 175,4 | 153,0 |
| 81 | 28 | 34,2 | 173,2 | 50,9 | 34,4 | 173,3 | 51,0 | 34,8 | 185,2 | 55,0 |
| 82 | 28 | 33,0 | 174,3 | 199,5 | 33,2 | 174,4 | 200,0 | 33,0 | 177,3 | 202,0 |
| 83 | 28 | 33,4 | 177,5 | 178,0 | 33,3 | 177,6 | 179,0 | 33,4 | 182,5 | 180,0 |
| 84 | 28 | 33,1 | 186,3 | 114,0 | 33,2 | 186,3 | 114,0 | 33,4 | 184,3 | 114,0 |
| 85 | 28 | 34,3 | 174,4 | 154,0 | 34,7 | 174,1 | 153,0 | 34,3 | 172,3 | 154,0 |
| 86 | 28 | 33,9 | 181,6 | 115,0 | 33,8 | 181,8 | 115,0 | 33,9 | 186,0 | 116,0 |
| 87 | 28 | 33,7 | 174,9 | 146,0 | 33,7 | 174,9 | 145,0 | 33,9 | 173,7 | 145,0 |
| 88 | 28 | 35,0 | 184,3 | 80,7 | 35,2 | 184,7 | 80,6 | 35,2 | 185,5 | 80,0 |
| 89 | 28 | 35,6 | 167,6 | 161,0 | 36,5 | 167,6 | 162,0 | 35,6 | 168,6 | 161,0 |
| 90 | 28 | 33,4 | 179,9 | 178,0 | 33,8 | 180,0 | 177,0 | 33,3 | 180,9 | 178,0 |
| 91 | 28 | 33,3 | 181,0 | 174,0 | 34,1 | 180,8 | 175,0 | 33,3 | 181,0 | 174,0 |
| 92 | 28 | 36,7 | 200,7 | 129,0 | 37,0 | 200,3 | 130,0 | 36,7 | 207,0 | 130,0 |
| 93 | 28 | 34,2 | 186,6 | 186,0 | 34,9 | 187,0 | 186,0 | 34,1 | 184,9 | 186,0 |
| 94 | 28 | 37,1 | 182,3 | 129,0 | 38,3 | 182,5 | 128,0 | 38,3 | 183,2 | 129,0 |
| 95 | 28 | 30,6 | 181,5 | 177,0 | 31,4 | 181,7 | 176,0 | 30,6 | 185,0 | 175,0 |
| 96 | 28 | 35,0 | 165,6 | 182,0 | 36,2 | 165,4 | 182,0 | 35,3 | 166,6 | 184,0 |
| 97 | 28 | 34,9 | 168,9 | 171,0 | 35,2 | 169,0 | 171,0 | 34,5 | 165,5 | 175,0 |
| 98 | 28 | 33,8 | 186,3 | 59,4 | 33,9 | 186,6 | 59,2 | 34,5 | 188,0 | 61,0 |
| 99 | 28 | 34,2 | 186,2 | 198,0 | 34,5 | 186,5 | 198,0 | 34,0 | 186,1 | 195,0 |
| 100 | 28 | 34,4 | 181,7 | 157,0 | 35,3 | 181,4 | 157,0 | 34,9 | 184,5 | 161,0 |
