# Supplementary material for: Calibration Markers for Digital Templating in Total Hip Arthroplasty
Source: PLoS One. 2015 Jul 13;10(7):e0128529. doi: 10.1371/journal.pone.0128529 (PMC4500467; doi:10.1371/journal.pone.0128529)

**S1 Text:** **Derivation of formula 4.**

Introduction: In a cartesian coordinate system the plane of projection is the *xy*-plane, and the source (or focus) F of the X-ray beam is located at height h over the origin O = (0; 0) of the plane of projection, i.e. F = (0; 0; h). The projected object, either a round disc or a sphere, is centered at a horizontal distance *x_0_* from the focus and at height *z_0_* over the plane of projection. The disc lies horizontally in a coronal plane, parallel to the *xy*-plane. The X-rays emanating from F and passing through the object determine a cone whose intersection with the *xy*-plane gives the projected image of the object (i.e. radiograph).

Projecting a disk: Figure 1 shows the projection of a single point P at height *z_0_* and horizontal distance s from the focus, in the vertical plane containing P and the z-axis. The projected point is P’. The triangles PFQ and P’FO are similar, described by formula:

(1)
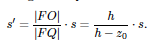


Therefore, the projected image of any object at height *z_0_* is stretched by the magnification factor:

(2)
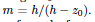
.

Projecting a sphere: A sphere of the radius *r* is centred at height *z_0_* over the plane of projection and at a horizontal distance x0 from F. By rotating the *xy*-coordinates, the centre is located at the point (*x_0_*; *0*; *z_0_*). For symmetry reasons, the major axis of the projected ellipse is on the x-axis. The corresponding *xz*-plane is shown in Figure 2 a. The major axis of the projected ellipse is given by the distance ⏐P’Q’⏐. Following Pythagoras’ theorem, the lengths of ⏐FC⏐ and ⏐FB⏐ are:

(6)
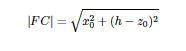
and

(7)
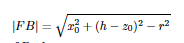
.

In Figure 2 b the detailed view of the tangential X-rays along the sphere is given. The right-angled triangle QBC is similar to the triangle QSF, implying:

(8)
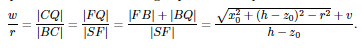
and

(9)
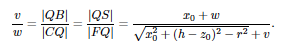
.

Equation 8 can be solved for *v*:

(10)
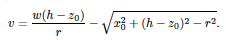


In combination with equation 9 we derive:

(11)
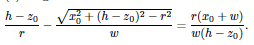


This equation can easily be solved for w by first multiplying with *rw* • (h - *z_0_*) and then collecting terms:

(12)
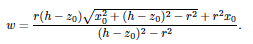


Similar, *w** can be derived from the triangles PAC and PSF:

(13)
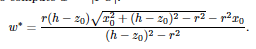


Summing equations 12 and 13 the length of ⏐PQ⏐ is:

(3)
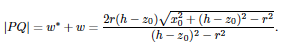


In combination with equation 2, the projected major axis of a sphere is:

(4)
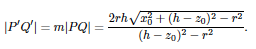

Supplement: S1 Text — (DOCX) [file pone.0128529.s004.docx]
