# Supplementary material for: Calibration Markers for Digital Templating in Total Hip Arthroplasty
Source: PLoS One. 2015 Jul 13;10(7):e0128529. doi: 10.1371/journal.pone.0128529 (PMC4500467; doi:10.1371/journal.pone.0128529)
Supplement: S2 Text — (PDF) [file pone.0128529.s005.pdf]

Formulae for

"Calibration markers for digital templating in total hip arthroplasty"

by C. K. Boese et al.

$$s' = \frac{|FO|}{|FQ|} \cdot s = \frac{h}{h - z_0} \cdot s \tag{1}$$

$$m = \frac{h}{h - z_0} \tag{2}$$

$$|PQ| = w^* + w = \frac{2r(h - z_0)\sqrt{x_0^2 + (h - z_0)^2 - r^2}}{(h - z_0)^2 - r^2} \tag{3}$$

$$|P'Q'| = m|PQ| = \frac{2rh\sqrt{x_0^2 + (h - z_0)^2 - r^2}}{(h - z_0)^2 - r^2} \tag{4}$$

$$\text{calibration factor} = \frac{\text{projected diameter}}{\text{true diameter}} \cdot 100 \tag{5}$$

$$|FC| = \sqrt{x_0^2 + (h - z_0)^2} \tag{6}$$

$$|FB| = \sqrt{x_0^2 + (h - z_0)^2 - r^2} \tag{7}$$

$$\frac{w}{r} = \frac{|CQ|}{|BC|} = \frac{|FQ|}{|SF|} = \frac{|FB| + |BQ|}{|SF|} = \frac{\sqrt{x_0^2 + (h - z_0)^2 - r^2} + v}{h - z_0} \tag{8}$$

$$\frac{v}{w} = \frac{|QB|}{|CQ|} = \frac{|QS|}{|FQ|} = \frac{x_0 + w}{\sqrt{x_0^2 + (h - z_0)^2 - r^2} + v} \tag{9}$$

$$v = \frac{w(h - z_0)}{r} - \sqrt{x_0^2 + (h - z_0)^2 - r^2} \tag{10}$$

$$\frac{h - z_0}{r} - \frac{\sqrt{x_0^2 + (h - z_0)^2 - r^2}}{w} = \frac{r(x_0 + w)}{w(h - z_0)} \tag{11}$$

$$w = \frac{r(h - z_0)\sqrt{x_0^2 + (h - z_0)^2 - r^2} + r^2 x_0}{(h - z_0)^2 - r^2} \tag{12}$$

$$w^* = \frac{r(h - z_0)\sqrt{x_0^2 + (h - z_0)^2 - r^2} - r^2 x_0}{(h - z_0)^2 - r^2} \tag{13}$$
